# Kidney transplantation with donors in severe acute kidney injury. Should we use these organs? Retrospective Case Series

**DOI:** 10.1590/2175-8239-JBN-2018-0264

**Published:** 2019-09-02

**Authors:** Guilherme Palhares Aversa Santos, Luis Gustavo Modelli de Andrade, Mariana Farina Valiatti, Mariana Moraes Contti, Hong Si Nga, Henrique Mochida Takase

**Affiliations:** 1 Universidade Estadual Paulista, Faculdade de Medicina de Botucatu, Departamento de Clínica Médica, Botucatu, SP, Brasil.

**Keywords:** Kidney Transplantation, Tissue Donors, Delayed Graft Function, Acute Kidney Injury, Transplante de Rim, Doadores de Tecidos, Função Retardada do Enxerto, Lesão Renal Aguda

## Abstract

**Introduction:**

The number of incident and prevalent patients on dialysis has increased, as well as the number of candidates for renal transplantation in Brazil, without a proportional increase in the number of organ donors. The use of expanded kidneys, as to renal function, may be an alternative to increase the supply of organs.

**Objective:**

to discuss the feasibility of using expanded kidneys for renal function, which are in severe acute renal injury.

**Methods:**

All cases of renal transplantation of deceased donors performed at the Hospital das Clínicas de Botucatu of UNESP, from January 2010 to June 2018, totaling 732 cases were evaluated. Cases with final donor creatinine greater than 6 mg/dL were selected.

**Results:**

four patients were selected, of whom all donors were in severe acute kidney injury (AKI). These donors presented rhabdomyolysis as a probable cause of severe AKI, were young, with no comorbidities and had decreased urinary volume in the last 24 hours. The clinical evolution of all the recipients was satisfactory, with a glomerular filtration rate after transplantation ranging from 48 to 98 mL/min/1.73 m^2^.

**Conclusion:**

this series of cases shows the possibility of using renal donors in severe AKI, provided the following are respected: donor age, rhabdomyolysis as the cause of AKI, and implantation-favorable biopsy findings. Additional studies with better designs, larger numbers of patients and longer follow-up times are needed.

## INTRODUCTION

In recent years, the number of incident and prevalent dialysis patients has increased, as has the number of renal transplant candidates in Brazil,^1^ without a proportional increase in the number of organ donors. The use of renal donors with expanded criteria is a measure that may help in solving this problem and has been increasingly adopted.^2^ Kidneys of donors with acute kidney injury (AKI) - KDIGO III, considered expanded kidneys as to renal function, are frequently discarded³ but its use may contribute to a greater supply of organs.

The objective of this series of cases was to discuss the feasibility of expanded kidneys with severe acute renal injury being used for renal function.

## METHOD

This is an observational, retrospective, case-series study. All cases of renal transplantation performed at the Hospital das Clínicas of UNESP from January 2010 to June 2018 were reviewed. Our goal was to evaluate the most severe and more challenging cases of AKI donors for the transplantation under the clinical viewpoint, in this eight-year period, we selected all patients transplanted from a deceased donor with a final creatinine level greater than 6 mg/dL. This creatinine cutoff value was based on the retrospective study carried out by Heilman et al., in which the maximum final creatinine of donors with AKI was 6.29 mg/dL.^4^

## RESULTS

A total of 732 transplant recipients from deceased donors were found in the period, of which 4 fulfilled the selection criteria. Clinical and laboratory data are described below.

Case 1. Female, 44 years old, having granulomatosis with polyangeitis as baseline disease, peritoneal dialysis for 2 years and 11 months, residual diuresis of 600 mL, antibody reactivity panel (ARP) of 0. Donor: female, 37 years old, cause of death, traumatic HSA after severe head injury, without comorbidities, three days of hospitalization in Intensive Care Unit (ICU), using Clavulanic acid to treat an infective pulmonary focus and noradrenaline 1.8 mcg/kg/min, diuresis of 300 mL in the past 24 hours, initial creatinine of 1.1 mg/dL, final creatinine of 7.4 mg/dL, CPK of 8,688 U/L, Kidney Donor Profile Index (KDPI) of 46%. Uptake renal biopsy with signs of ATN. Cold ischemia duration (CID) of 23 hours and 30 minutes, induction therapy with methylprednisolone 500 mg and thymoglobulin 3 mg/kg and maintenance therapy with tacrolimus, everolimus and prednisone. The transplantation occurred without problems, with no urologic or vascular complications. The patient had delayed graft function (DGF) in the postoperative period, with progressive improvement of nitrogenous waste and urinary output from the second week after transplantation. She was discharged from the hospital on the 30th postoperative day. Currently, with two years and eight months of transplantation, she has creatinine of 1.0 mg/dL and TFG (by CKD-epi) of 67 mL/min/1.73 m^2^.

Case 2. Male, 38 years old, unknown baseline disease, without residual diuresis, in renal replacement therapy for 9 years and 5 months, under hemodialysis, ARP of 0. The donor is the same as that of Case 1 patient. TIF of 25 hours and 5 minutes, induction therapy with methylprednisolone 500 mg and thymoglobulin 3 mg/kg and maintenance therapy with tacrolimus, everolimus and prednisone. Transplantation occurred without intercurrences, with no urologic or vascular complications. He developed DGF, with partial improvement of the waste and urine output from the 14th day of hospitalization. On account of prolonged DGF, he was biopsied at the 18th postoperative day, with pathology compatible with ATN. He was discharged in the 28^th^ postoperative day. C urrently, with the same time of transplantation as the patient of case 1, he has a GFR of 58 mL/min/1.73 m^2^.

Case 3. Female, 52 years old, unknown baseline disease, on hemodialysis for 4 years and 11 months, residual diuresis of 500 mL, ARP of 34%. The donor, A 43-year-old male smoker, had acute subdural hematoma after traumatic head injury as the cause of death, two days of ICU, using noradrenaline 0.8 mcg/kg/minute, vasopressin 6 IU/hour, diuresis in the last 24h of 120 mL, initial creatinine of 1.1 mg/dL and final creatinine of 12.2 mg/dL, CPK of 58,124 U/L, 54% KDPI. The uptake renal biopsy presented ATN. TIF of 22 hours and 46 minutes, induction therapy with methylprednisolone 500 mg and thymoglobulin 4.5 mg/kg (divided into 1.5 mg/kg initiated on anesthetic induction, and then 1 mg/kg for 3 more days) and therapy with tacrolimus, mycophenolate and prednisone. Surgery occurred without complications, with no vascular or urologic complications. Patient evolved with DGF, being biopsied in the 13^th^ postoperative due to prolonged DGF, histopathologically compatible with ATN. There was an increase in urine output and progressive waste formation from the 21st day of hospitalization. He presented several infectious intercurrences (UTI, esophageal moniliasis, cytomegalovirus infection); he was discharged from the hospital in the 46th postoperative day. Currently, after 5 months and 13 days of transplantation, he has a creatinine level of 0.8 mg/dL and GFR of 98 mL/min/1.73 m^2^.

Case 4. Male, 35 years, unknown baseline disease for 1 year and 5 months, residual diuresis of 1000 mL, ARP of 0. Male donor, 35 years old, traumatic HI as cause of death, chronic alcoholic, 6 days of ICU, diuresis of 350 mL in the last 24 hours using noradrenalin 0.16 mcg/kg/min, initial creatinine of 1.1 mg/dL and final of 6.5 mg/dL, CPK of 11,922 IU/L, 33% KDPI. Uptake renal biopsy ATN-compatible. TIF of 20h33 minutes. Induction therapy with 500 mg of methylprednisolone and thymoglobulin 4.5 mg/kg, and maintenance with tacrolimus, mycophenolate and prednisone. Surgery occurred without complications, with no urologic or vascular complications. He evolved with DGF, underwent a biopsy on the 15th postoperative day with ATN findings. As of the 21^st^ postoperative day, there was a renal function improvement, and he was discharged on the 29^th^ POD. Currently, after 6 months and 12 days of transplantation, he has creatinine of 1.8 mg/dL and GFR of 48 mL/min/ 1.73 m^2^.

Table 1 depicts clinical and laboratory data from donors and recipients.

**Table 1:** Clinical and Laboratory Data from Donors and Recipients

	Cr (1)	Cr (2)	Cr (3)	Cr (4)	Cr (7)	Uptake biopsy	Donor age	C.P.K. (UI/mL)	ARP (%)	UO (mL)
Case 1	6.3	6.4	4.4	2	1.1	ATN	37	8688	0	300
Case 2	8.6	13.7	9.1	5.1	1.6	ATN	37	8688	0	300
Case 3	8.3	3.5	6.1	1.8	0.8	ATN	43	58124	34	120
Case 4	9.8	15.6	7.7	4.4	2.2	ATN	35	11922	0	350

Note: C.P.K: creatine phosphokinase. Cr (1): cr in the 1^st^ week, Cr(2): cr in the 2nd week, Cr(3): cr in the 3^rd^ week, Cr(4): cr in the 4^th^ week, Cr(7): cr in the 7^th^. UO:

## DISCUSSION

In this retrospective series of four cases, we evaluated the experience of a tertiary care hospital with kidney transplantation from donors in KDIGO III AKI and with final creatinine higher than 6 mg/dL.

All donors in this series are considered to be of the standard category according to the classic criteria of United Network for Organ Sharing (UNOS)^5^ expanded donors, but we can consider them to be borderline in renal function. Donors in this series were young, were on KDIGO III AKI and had renal biopsy of the ATN uptake (with no significant histological changes in the glomerular, tubule-interstitial and vascular compartments). The most likely renal insults were ischemic and nephrotoxic by rhabdomyolysis, with good prospects for recovery.

Regarding renal donors with altered creatinine, Kayler et al., in a retrospective study using the American transplant registry system, showed that, for standard criteria donors, the increase in serum creatinine in the donation was not associated with a decrease in survival graft, despite a higher percentage of DGF. In addition, for standard donors, the presence of final creatinine greater than 2 mg/dL increased the risk of organ rejection 7 fold,^3^ showing an underuse of these organs.

Boffa et al., In a retrospective study, analyzed the UK transplant registration system between 2003 and 2013. The presence of AKI in the donor increased the likelihood of kidney discarding, and the longer the stage of AKI, the greater the risk of not using the organ. In kidney donors with AKIN III AKI, the risk of organ rejection increased from 3 to 20 fold.^6^ There was little difference in graft survival comparing kidneys from donors with AKI and those without AKI (91% vs. 89%, *p* = 0.02). However, when compared with the survival of patients in the queue for transplantation or on dialysis, this small difference does not have clinical relevance. In this study, there was an association between donors with a higher stage of AKI (AKIN III) and a higher rate of non-functioning primary organs; however, the number of donors with AKIN III AKI was small in the sample studied.

Zheng et al., in a systematic review and meta-analysis of 2018 with 14 cohort studies, involving 15,345 donors, did not find a significant difference in relation to graft survival and in relation to primary non-functioning organs, when comparing kidney recipients from AKI donors with those without AKI.^7^ Graft survival was assessed within one to ten years after transplantation. In addition, GFR and episodes of acute rejection were similar between the groups. In patients with AKI, there was longer hospital stay and a higher incidence of DGF.

Heilman and colleagues retrospectively assessed the outcomes of 162 kidney recipients from AKI donors over a period of about 9 years. Of these recipients, 139 received kidneys considered standard according to UNOS. It is noteworthy that among these standard kidney donors, 50% were oligoanuric, 71% with AKIN III stage AKI and 21% required renal replacement therapy. The final maximum creatinine in this group of standard donors in AKI was 6.29 mg/dL. However, with the exception of DGF, which was higher in the group of patients with AKI, there was no difference in the incidence of acute rejection and graft survival in one year compared to patients who transplanted in the same period and received kidneys from donors who were not in AKI.^4^

Donor age, one of the criteria for considering an offered kidney as borderline, is well established as a condition that requires histological evaluation of the kidney to be implanted.^5^^,^ 8 This characteristic is also important to consider in cases of renal donation whose donor is in AKI, the age of the donor, rather than the final creatinine value has a greater association with outcome and worsening renal function in 6 months.^9^

The histological assessment of the AKI kidneys is fundamental, as it shows their viability before implantation. The absence or discrete histological lesions correlate with good graft survival in one year, favoring the use of the organ offered; while the presence of moderate to severe lesions correlates with poor graft survival, making transplantation unwise.^10^^,^ 11 Kyler and colleagues reviewed 597 renal donor biopsies performed between 1987 and 2006; arteriosclerosis, or moderate arteriolosclerosis, defined as a decrease in vascular lumen equal to or greater than 25%, were independently associated with poorer graft survival at one, three, and five years for both standard and marginal donors.^10^

Regarding the clinical course of AKI, these three donors were in a clear phase with oliguria, and the possibility of acute renal support could be considered, except for the condition of brain death. Such a clinical evolution could have led to organ rejection in many transplantation centers.

Tomita et al. Reported their experience with eight cardiac arrest donors who presented KDIGO III AKI, with creatinine greater than 10 mg/dL, prior to renal uptake. However, unlike the present series of cases, at the time of collection, patients already had adequate urine output and recovery of renal function. Most of these patients had good renal function after the first month of transplantation, as occurred with our patients, and adequate long-term graft survival.^12^

It is possible to argue that all three donors in this study had AKI for rhabdomyolysis. In a retrospective observational study carried out by Chen et al., they analyzed 30 patients diagnosed with KDIGO III AKI kidneys during rhabdomyolysis, compared to 90 patients who received standard kidneys, and they reported that there was no statistical difference in relation to creatinine and GFR at months 2, 6, 12 and 24; there was also no difference in the incidence of rejection,^13^ showing good graft function in the long term.

All patients reported in this series had DGF, as expected, since the percentage of graft delay in renal donors with AKI is high.^6^^,^ 7 ^,^ 8 ^,^ 9 The main risk factor and probable etiological factor for DGF is the KDIGO III AKI diagnosis validity prior to donation. Other risk factors include the long dialysis time prior to transplantation in the first case and the 25-hour CID in the third case.

The results and outcomes of this series of cases should be considered according to the nature of this study, that is, with limited power of association and generalization. Studies with more patients and longer follow-up are necessary.

## CONCLUSION

This case series shows the possibility of using KDIGO III AKI donor kidneys with high final creatinine; observing the donor age conditions, kidney biopsy without histological lesions or discrete lesions and ischemic AKI with rabdomyolysis. Further studies, with better designs and larger numbers of patients are needed in order to assess the true possibility of using these kidneys.
